# Kinesio Taping^®^ is not better than placebo in reducing pain and disability in patients with chronic non-specific low back pain: a randomized controlled trial

**DOI:** 10.1590/bjpt-rbf.2014.0128

**Published:** 2015-10-09

**Authors:** Maurício A. Luz, Manoel V. Sousa, Luciana A. F. S. Neves, Aline A. C. Cezar, Leonardo O. P. Costa

**Affiliations:** 1 Departamento de Fisioterapia, Universidade Paulista (UNIP), São Paulo, SP, Brazil; 2 Departamento de Fisioterapia, UNIP, Jundiaí, SP, Brazil; 3 Programa de Mestrado e Doutorado em Fisioterapia, Universidade Cidade de São Paulo (UNICID), São Paulo, SP, Brazil; 4 Musculoskeletal Division, The George Institute for Global Health, Sydney, Australia

**Keywords:** physical therapy, kinesio taping, tape, lower back pain, rehabilitation

## Abstract

**Background:**

*Kinesio Taping* ^®^ has been widely used in clinical practice. However, it is unknown whether this type of tape is more effective than placebo taping in patients with chronic lower back pain.

**Objective:**

To compare the effectiveness of *Kinesio Taping* ^®^ in patients with chronic non-specific low back pain against a placebo tape and a control group.

**Method:**

This is a 3-arm, randomized controlled trial with a blinded assessor. Sixty patients with chronic non-specific low back pain were randomized into one of the three groups: *Kinesio Taping* ^®^ group (n=20), Micropore^®^ (placebo) group (n=20) and control group (n=20). Patients allocated to both the *Kinesio Taping* ^®^ group and the placebo group used the different types of tape for a period of 48 hours. The control group did not receive any intervention. The outcomes measured were pain intensity (measured by an 11-point numerical rating scale) and disability (measured by the 24-item Roland Morris Disability Questionnaire). A blinded assessor measured the outcomes at baseline, 48 hours and 7 days after randomization.

**Results:**

After 48 hours, there was a statistically significant difference between the *Kinesio Taping* ^®^ group versus the control group (mean between-group difference = -3.1 points, 95% CI=-5.2 to -1.1, p=0.003), but no difference when compared to the placebo group (mean between-group difference= 1.9 points, 95% CI=-0.2 to 3.9, p=0.08). For the other outcomes no differences were observed.

**Conclusions:**

The *Kinesio Taping* ^®^ is not better than placebo (Micropore^®)^ in patients with chronic low back pain.

## Introduction

Low back pain is a serious worldwide health problem^1^^,^ 2 and has been quoted as the major cause of disability around the world^3^^,^ 4. In Brazil, spinal pain (cervical, thoracic and lumbar) was considered the second most prevalent complaint, affecting approximately 13.5% of the population^5^. It is estimated that globally 39% of the population will have at least one episode of back pain throughout their lives^6^. In episodes of pain greater than 12 weeks (classified as chronic lower back pain^2^), the prognosis is unfavorable^2^ and is highly associated with high treatment costs and work absenteeism^7^.

A technique widely used today to assist in the treatment of various musculoskeletal conditions is an elastic tape, called *Kinesio Taping* ^®2,8,9^. The technique was developed in the 1970s in Japan by Kase et al.^10^ and consists of tape applied to the skin. This tape has elasticity in the longitudinal direction with an elongation of 40% to 60% from its resting length^10^. The effects of the *Kinesio Taping* ^®^ described by its creators included: changes in muscle activation, reduction of pain, joint repositioning and reduction of abnormal muscular tension^10^^,^ 11. The use of this technique is widespread in the sports area^12^^,^ 13 and is also common in clinical practice^14^. In the 2008 Olympics, kilometers of *Kinesio Taping* ^®^ tapes were donated to the delegations of 58 countries, increasing its exposure and curiosity in the use of the tape^13^^,^ 15. At the 2012 Olympics, it was noted that the technique had been used by more than 80 delegations^12^.

In recent years, an increasing number of studies using *Kinesio Taping* ^®^ for pain relief have been conducted^16^^-^ 20; however only three of them used the *Kinesio Taping* ^®^ in the treatment of non-specific chronic lower back pain^16^^,^ 17 ^,^ 19. Parreira et al.^16^ compared two forms of application of this tape. One group of patients received the tape as described in the official manual of *Kinesio Taping* ^®^ Association International^21^, with tension between 10-15%, generating circumvolutions which are winding movements of the tape around a body part, and in a second group, the tape was applied without any tension to avoid circumvolutions. The authors found no significant difference between groups, which raises the question about the need of circumvolutions when applying the tape. Castro Sánchez et al.^17^compared the *Kinesio Taping* ^®^ with a placebo group and the results showed that, although statistically significant for the pain and disability outcomes, the effects were so small that authors did not considered clinically important in relation to the placebo application results. Paoloni et al.^19^used the *Kinesio Taping* ^®^ combined with therapeutic exercises. Although the authors observed a decrease in the EMG activity in the paraspinal muscles in patients who underwent the *Kinesio Taping* ^®^ methods, the results were not statistically significant for the pain when compared to patients who only underwent therapeutic exercises. Studies have been compiled in systematic reviews^8^^,^ 13 ^,^ 22 ^-^ 24 and meta analysis^25^, and concluded, based on recommendations of the Grading of Recommendations, Assessment, Development, and Evaluation (GRADE) from the Cochrane Collaboration^26^, that there is low quality evidence for the application of *Kinesio Taping* ^®^ in patients with lower back pain. The term "low quality evidence" means that further studies may or may not eventually alter the conclusions of these reviews^26^. Therefore, new randomized controlled trials with high methodological quality trials need to be conducted.

To date, there is no information on the effects of *Kinesio Taping* ^®^ due to the lack of studies that compared a group of patients receiving to this intervention versus a control group that did not receive the intervention. Furthermore, in the presence of some beneficial effect, it was unclear whether this effect was due to the intervention or simply due to a placebo effect. Therefore, the aim of this study was to compare the effectiveness of applying *Kinesio Taping* ^®^, Micropore^®^ taping (placebo therapy) and a control group with no taping on the outcomes of pain and disability in patients with chronic non-specific low back pain. The observed results were obtained 48 hours and seven days following the application of the different taping methods to the 2 groups for 48 hours. Our hypothesis was that patients who received *Kinesio Taping* ^®^ would demonstrate greater clinical improvements when compared to patients allocated to the placebo and control groups.

## Method

### Study design

A three-arm, randomized controlled trial with a blinded assessor was conducted. This study was approved by the Research Ethics Committee of *Universidade Paulista* (UNIP), São Paulo, SP, Brazil (number 304 408) and prospectively registered at ClinicalTrials.gov (NCT0200766). All patients signed a consent form before the inception of the study.

### Study location

The present study was conducted at the Physical Therapy Clinic of UNIP, Campus Jundiaí, SP, Brazil and at private physical therapy clinics in Campo Limpo Paulista City, SP, Brazil, from August to December of 2013.

### Subjects

Subjects of both sexes and between 18 and 80 years of age were included. They were referred to physical therapy service by a physician for treatment of chronic non-specific low back pain (back pain of mechanical origin, apparently without a defined cause, for at least 12 weeks duration)^27^. In addition, participants had not had any physical therapy treatment in the past six months and had never used *Kinesio Taping* ^®^. Exclusion criteria were: presence of skin diseases; contraindication due to the use of the tape, serious spinal pathologies such as a tumor, an inflammatory disease or fracture; nerve root compromise; pregnancy; subjects who had physical therapy treatment in the past six months, and subjects who had used or had prior knowledge of the *Kinesio Taping* ^®^ method. Nerve root compromise was tested through clinical examination involving tests of strength, sensitivity and reflexes following the recommendations of the *European Guidelines for the Management of Patients with Back Pain* 1.

### Randomization and interventions

After the baseline assessment, participants were referred to the physical therapist responsible for the interventions. An independent researcher, who was not involved in the recruitment of the participants, executed a randomization program on a computer to assign each individual to a specific test group. Each participant received a sealed, opaque envelope that revealed their assigned group. The groups were:

1. *Kinesio Taping* ^®^ Group: the *Kinesio* ^®^ Tex Classic beige tape was used and applied over the erector spinae muscle with 10-15% of tension in the stretched position, as described in the official manual of *Kinesio Taping* ^®^ Association International^21^.
2. Micropore^®^ Group: the Micropore^®^ 3M^®^tape beige tape was used and applied over the erector spinae muscle in the stretched position.
3. Control Group: this group did not receive any tape intervention.

The participants allocated to the *Kinesio Taping* ^®^ and Micropore^®^ intervention groups received the tape application once and the tape remained in place for 48 hours, following the instructions of the *Kinesio Taping* ^®^ Association International to minimize the risk of allergies or skin damage. After the application, the subjects were instructed to remove the tape if they had any allergic reaction due to the tape, and in cases where the tapes became loose and began to fall off, the subjects were instructed to report when the tape fell off or was removed to the evaluators at the next evaluation which was when the tape was supposed to be removed (then the tape was re-applied). The application was performed by a physical therapist who had over nine years of clinical experience in treating patients with lower back pain and had formal training in the application of *Kinesio Taping* ^®^ (level KT3) of the *Kinesio Taping* ^®^ Association International). After randomization, instructions about the characteristics and expected effects of the *Kinesio Taping* ^®^, such as pain relief, were given to the *Kinesio Taping* ^®^ and Micropore^®^ groups. During the application of the tapes to the groups receiving intervention, subjects were positioned backwards to the physical therapist applying the tape and the distal part of the tape was attached to the posterior superior iliac spine; subjects were then asked to bend the trunk forward until they were in a comfortable flexed position. The tapes were applied over the erector spinae muscles bilaterally moving upward to the 8^th^thoracic vertebrae. The therapists took about 1-2 minutes to apply the tapes. The control group did not receive any intervention. All subjects were scheduled to begin the physical therapy treatment at the end of the test period (i.e. 7 days after randomization).

### Evaluation and instruments

The evaluations were performed at baseline, 48 hours and seven days after randomization, by a blinded evaluator who was unaware of which group the subjects were allocated to. The initial assessment occurred in the clinic and the 48-hour evaluation was conducted by telephone. The evaluation at seven days, in most cases, was carried out in the clinic when the patient returned to start the conventional physical therapy treatment. If the patient missed the day to start the conventional treatment, the evaluation was also conducted by telephone. It was impossible to blind the therapist to the different tapes applied. Furthermore, due to the presence of a group with no taping, the subjects were not blinded to the treatment they received.

The outcomes measured were pain intensity and disability. Pain intensity was evaluated using a pain numeric rating scale^28^, consisting of 11 items, with 0 being "no pain" and 10 the "worst possible pain". Disability was assessed using the Brazilian version of the Roland Morris Disability Questionnaire (RMDQ)^29^, which contains 24 items related to daily activities that might be impaired due to low back pain where each affirmative answer corresponds to a point on the scale. The final score of the RMDQ was determined by summation of the values obtained: the higher the score, the greater the disability. These scales were cross culturally adapted and tested for the Brazilian Portuguese language^30^^,^ 31.

### Statistical analysis

The study was designed to detect a clinically important difference for the outcomes of pain and disability^32^. For pain intensity, a difference of two points was calculated, as measured by the Portuguese version of the Numerical Pain Rating Scale (with a standard deviation estimated at 2.05 points), and three points for disability assessed by Roland Morris Disability Questionnaire^29^ (with a standard deviation estimated at 5.1 points). A α=0.05, a statistical power of 80% and a sample loss of 15% were considered. The sample size calculation resulted in a sample of 20 participants per group, totaling 60 subjects.

A double entry of data was performed, and the analysis followed the principles of intention to treat. Data normality was tested by visual inspection of the histograms, and all data were normally distributed. The confidence interval was set to 95% for all analyses. Estimates of average effects (i.e. between-group differences) for all outcomes were calculated using linear mixed models. These longitudinal models of analyses incorporate terms for treatment groups (*Kinesio Taping* ^®^, placebo and control), time (baseline, 48 hours, and seven days post-randomization), and interactions terms of group versus time. The regression coefficients from the interaction group versus time were equivalent to the estimates of the between group differences of the effects of interventions. The post-hoc analyses for multiple comparisons were performed. Data were analyzed using the SPSS 19 for Windows software.

## Results

**Figure 1:**
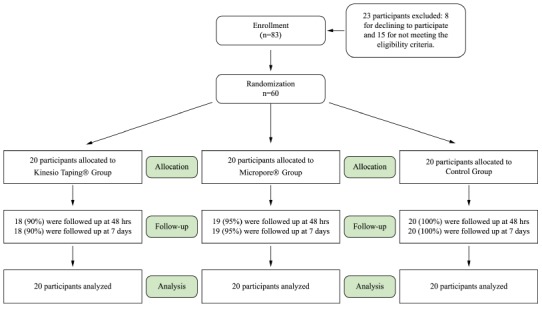
Flow diagram of participants throughout the study.

The recruitment of the subjects from the physical therapy clinics was conducted between August and December of 2013. Eighty-three patients with chronic low back pain were enrolled; of these, 23 were excluded because they did not meet the eligibility criteria. Eight patients declined to participate, nine had previously undergone back surgery, five were excluded because of nerve root compromise and one was excluded due to the presence of psoriasis (Figure 1). 

This study included 60 participants with chronic non-specific low back pain, randomly allocated into three groups; a *Kinesio Taping* ^®^ group (11 women and nine men, mean age of 44.3 years, SD=15.0); a Micropore^®^ group (13 women and seven men, mean age of 50.1 years, SD=17.5); and a control group (17 women and three men, mean age of 48.1 years, SD=13.4). The demographic characteristics of the sample are shown in Table 1. 

**Table 1.** Baseline characteristics of subjects with chronic low back pain who received Kinesio Taping, micropore taping or had no intervention.

**Variable**	**Participants**		
	**Kinesio Taping**® ** (n=20)**	**Micropore® Taping (n=20)**	**Control (n=20)**
Age (y)	44.3 (15.0)	50.1 (17.5)	48.1 (13.4)
Gender			
Male	9 (45)	7 (35)	3 (15)
Female	11 (55)	13 (65)	17 (85)
Duration of low back pain (mo)*	76.4 (61.6)	49.6 (42.4)	82.2 (63.4)
Weight (kg)	72.5 (7.1)	74.9 (15.7)	79.7 (20.9)
Height (m)	1.67 (0.1)	1.66 (0.1)	1.65 (0.1)
Body mass index (kg/m2)	26.0 (3.0)	27.1 (4.7)	30.3 (7.4)
Marital status			
Single	2 (10)	3 (15)	1 (5)
Married	16 (80)	13 (65)	18 (90)
Divorced	2 (10)	1 (5)	1 (5)
Widower	0 (0)	3 (15)	0 (0)
Academic level			
Primary education	10 (50)	10 (50)	13 (65)
Secondary education	6 (30)	6 (30)	4 (20)
Academic education	3 (15)	4 (20)	3 (15)
MBA	1 (5)	0 (0)	0 (0)
Income (in minimum wages)	3.3 (2.4)	3.6 (3.1)	3.1 (2.2)
Physical therapy treatment			
Yes	11 (55)	11 (55)	9 (45)
Use of medication			
Yes	9 (45)	6 (30)	5 (25)
Pain intensity (0-10)	6.6 (1.2)	6.7 (1.6)	6.1 (2.1)
Disability (0-24)	12.9 (5.6)	12.2 (6.5)	11.8 (6.5)


The categorical variables are expressed as n (%), and the continuous variables are expressed as mean (SD). *Expressed as median (IQR).

Regarding the use of medications, in the *Kinesio Taping* ^®^ group nine patients were taking medication as follows: four were taking painkillers, three were taking muscle relaxants and two were taking anti inflammatory drugs. In the Micropore^®^ group, six patients were using medication as follows: one patient was taking analgesics, three were taking muscle relaxants and two were taking anti-inflammatory drugs. In the control group, five patients were using medication as follows: two were taking painkillers, one was taking a muscle relaxant and two were taking anti-inflammatory drugs.

From the *Kinesio Taping* ^®^ group, two participants (10%) abandoned the study and missed the evaluation phases of 48 hours and seven days. From the Micropore^®^ group, one participant (5%) abandoned the study and missed the 48-hour and seven days evaluation. From the control group, none of the participants abandoned the study.

Table 2 shows the mean and standard deviation of the pain intensity^28^ and disability^29^. Table 3 shows the between-group analysis for all comparisons. A statistically significant difference was observed between the *Kinesio Taping* ^®^ group and control group for the disability outcome (mean difference of -3.1 points; 95% CI=-5.2 to -1.1) at the 48-hour follow up. No differences were detected between the *Kinesio Taping* ^®^ and placebo groups for all the outcomes analyzed.

**Table 2.** Means (SD) at baseline and 48 hours and seven-day follow-ups for subjects with chronic low back pain who received Kinesio Taping, Micropore taping or had no intervention.

**Outcome**	**Interventions**											
	**Baseline**			**Follow-up 48 hours**			**Follow-up 7 days**		
	**Kinesio Taping® Group**	**Micropore® Taping Group **	**Control Group **	**Kinesio Taping® **	**Micropore® Taping Group **	**Control Group **	**Kinesio Taping®**	**Micropore® Taping Group**	**Control Group**
Pain	6.6	6.7	6.1	4.9	5.1	5.4	5.8	6.3	5.5
(0-10)	(1.2)	(1.6)	(2.1)	(2.6)	(2.7)	(2.6)	(1.3)	(2.0)	(1.9)
Disability	12.8	12.2	11.8	8.6	9.4	10.6	9.6	10.2	10.3
(0-24)	(5.6)	(6.5)	(6.5)	(5.6)	(6.7)	(6.9)	(5.6)	(7.4)	(6.6)


Data expressed as mean and standard deviation (SD).

**Table 3.** Between-group differences at 48 hours and 7-days after randomization for subjects with chronic low back pain who received Kinesio Taping, Micropore taping or had no intervention.

**Outcome**	**Difference between interventions Adjusted Mean Difference (95% CI)**											
	**Baseline to Follow-up at 48 hours (95% CI), p**						**Baseline to Follow-up at 7 days (95% CI), p**					
	**Kinesio vs Micropore**	**p**	**Kinesio vs Control**	**p**	**Micropore vs Control**	**p**	**Kinesio vs Micropore**	**p**	**Kinesio vs Control**	**p**	**Micropore vs Control**	**p**
Pain	0.1	0.82	-1.0	0.09	-0.8	0.13	0.3	0.54	-0.2	0.76	0.2	0.75
(0-10)	(-1.0 to 1.2)		(-2.1 to 0.1)		(-1.9 to 0.3)		(-0.8 to 1.5)		(-1.3 to 0.9)		(-0-9 to 1.3)	
Disability	1.9	0.08	-3.1*	0.003	-1.3	0.22	1.7	0.11	-1.8	0.08	-0.1	0.89
(0-24)	(-0.2 to 3.9)		(-5.2 to -1.1)		(-3.3 to 0.8)		(-0.4 to 3.8)		(-3.9 to 0.2)		(-2.2 to 1.9)	


*Significant difference (p<0.05).

## Discussion

This study tested the effects of a single application of *Kinesio Taping* ^®^ compared with Micropore^®^ (placebo group) taping and a control group with no intervention in patients with chronic non-specific low back pain for the outcomes of pain intensity and disability. This is the first study that compared the *Kinesio Taping* ^®^ method with Micropore^®^ taping as a form of placebo therapy. The authors observed that, although the *Kinesio Taping* ^®^ group showed an improved disability score 48 hours after the application of the tape, the observed difference is so small that it could not be considered clinically important. All other statistical comparisons between groups showed no statistical significance. These findings raise a question regarding the use of *Kinesio Taping* ^®^ in clinical practice for patients with chronic non-specific low back pain since the effects observed (small) appears to be due to the placebo effect, regression to the mean, natural history and other possible confounders.

One of the strengths of this study is related to the recruitment of subjects. It has been shown that studies which recruited subjects seeking treatment for low back pain get more representative results than studies which recruited subjects from the community^33^. The limitations of our study included the fact that clinician were not blinded to the allocation of the participants to the groups - this was impossible due to the therapist's experience with the use of *Kinesio Taping* ^®^ - and that some of the seven-day assessments were conducted at the clinic while others were conducted by phone. This criterion was adopted to avoid a sample loss above 15%, which could interfere with the results due to attrition bias. Although the subjects allocated in the Micropore^®^ group were inclined to believe that they were using the *Kinesio Taping* ^®^ tape, the authors cannot consider this as a blinded study since the participants allocated to the control group received no intervention for the seven days. Participants from the control group were asked to avoid telling the assessor, at the time of the reassessment, whether they had or had not received taping. This allowed for the evaluator to remain blinded during the study. Finally, the authors observed that there was a higher proportion of painkiller users in the *Kinesio Taping* ^®^ group, which may have influenced the study results.

Although all participants showed some improvement (as described in Table 2), these differences could not be considered clinically important since none of these differences were greater than two points for the pain outcome and five points for the disability outcome, which were the cutoff points considered clinically significant^32^. 

When the pain outcome was analyzed between groups, no statistically significant difference was observed. However, this result should be considered with caution, since the effect size was approximately one point when the two groups that received intervention were compared with the control group. When the authors analyzed the *Kinesio Taping* ^®^ group versus the Micropore^®^ group, this difference was practically nil, which favors the hypothesis that the *Kinesio Taping* ^®^ is similar to the Micropore^®^ taping in the treatment of patients with chronic non-specific low back pain. In a study^19^ conducted using *Kinesio Taping* ^®^ associated with therapeutic exercises, where one group received the taping, a second group received the tape combined with therapeutic exercises, and a third group received only the therapeutic exercises, the results showed no difference among the groups. These results corroborate our study, since there was no statistically significant difference between the groups that received a taping intervention^16^.

In relation to the disability outcome, the difference was only statistically significant different when the *Kinesio Taping* ^®^ group was compared to the control group after 48 hours. However, the observed difference was too small and could not be considered clinically important. In addition, these differences were not observed at seven days. In another study^17^ that compared the application of *Kinesio Taping* ^®^ versus a placebo application the results were favorable for the *Kinesio Taping* ^®^ group for the outcomes pain and disability. The hypothesis for the difference observed might be related to how the taping was applied. For the *Kinesio Taping* ^®^ group, four strips with 25% of tension were superimposed, in a star format, to the point of greatest pain, while the placebo group received a single strip without tension in the transverse direction over the greatest point of pain. The difference in placement may have been more comfortable for the subjects that used more strips. These results showed the importance of studies focused on analyzing the different types of tape placement. Studies describing the electromyographic activity of muscles submitted to different tape and tension applications of *Kinesio Taping* ^®^ should also be encouraged.

Although systematic reviews^8^^,^ 13 ^,^ 22 ^-^ 24 do not recommend the use of *Kinesio Taping* ^®^ in clinical practice, the results of this study suggest that *Kinesio Taping* ^®^ was superior to no treatment for the disability outcome 48 hours after the application of the tape. For the pain outcome, although no statistically significant differences were found, the effect size was slightly higher in the groups using *Kinesio Taping* ^®^ and Micropore^®^ taping when compared to the control group. These results raise the hypothesis that subjects who received *Kinesio Taping* ^®^ or Micropore^®^ taping may remain more active and returned to their normal activities earlier, as it is recommended for patients with back pain^34^^,^ 35, than patients who did not receive any form of intervention. However these improvements are due to placebo effects only.

## Conclusion

The results showed that *Kinesio Taping* ^®^ showed similar results to Micropore^®^ taping in the outcomes investigated at 48 hours and at seven days after baseline testing. The *Kinesio Taping* ^®^ intervention was superior only when compared to the control group for the disability outcome at the 48-hour assessment. Therefore, the results of this study confirm that the therapeutic effects of the *Kinesio Taping* ^®^ are similar to the placebo effect. These results suggest that physical therapists should avoid this type of therapy.
